# Successful treatment of poison ivy dermatitis with upadacitinib

**DOI:** 10.1016/j.jdcr.2026.01.053

**Published:** 2026-02-09

**Authors:** Hansen Tai, Alice Tang, Jasmine Levine, Jordan Talia, Benjamin Ungar

**Affiliations:** a Department of Dermatology, SUNY Upstate Medical University College of Medicine, Syracuse, New York; b Department of Dermatology, Icahn School of Medicine at Mount Sinai, New York, New York

**Keywords:** allergic contact dermatitis, Janus kinase inhibitors, poison ivy, upadacitinib

## Introduction

Allergic contact dermatitis (ACD), including Toxicodendron contact dermatitis (TCD) from poison ivy, oak, and sumac, is a delayed hypersensitivity reaction to the catechol urushiol and commonly presents as an intensely pruritic papulovesicular eruption with potential for prolonged courses and more severe reactions upon re-exposure.^1^ Despite the prevalence of urushiol-containing plants in North America, current therapies are suboptimal. Topical and short-term oral corticosteroids are often inadequate for a complete clinical response, while long-term oral steroids are associated with numerous adverse effects such as weight gain and mood changes.^2^^,^^3^ Upadacitinib, a selective JAK1 inhibitor approved for atopic dermatitis (AD), modulates cytokine mediated inflammation by targeting JAK-STAT signaling,^4^^,^^5^ and is increasingly considered for conditions with type IV, T-cell–driven immunopathology.^6^^,^^7^ Given the mechanistic overlap between AD and ACD/TCD (T-cell activation and cytokine signaling), JAK inhibition represents a biologically plausible targeted approach for refractory disease. Here, we report a middle-aged patient who experienced rapid and durable improvement of severe TCD with upadacitinib. This case highlights the potential role of JAK1 inhibition in TCD and adds to emerging evidence supporting modulation of the JAK pathway in contact dermatitis.

## Case

**Fig 1:**
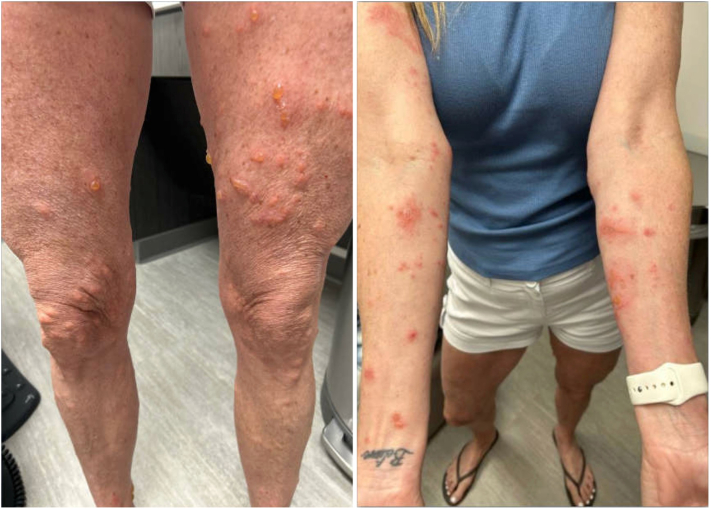
Clinical features of toxicodendron contact dermatitis (TCD) characterized by linear erythematous papules and blisters on exposed extremities.

**Fig 2:**
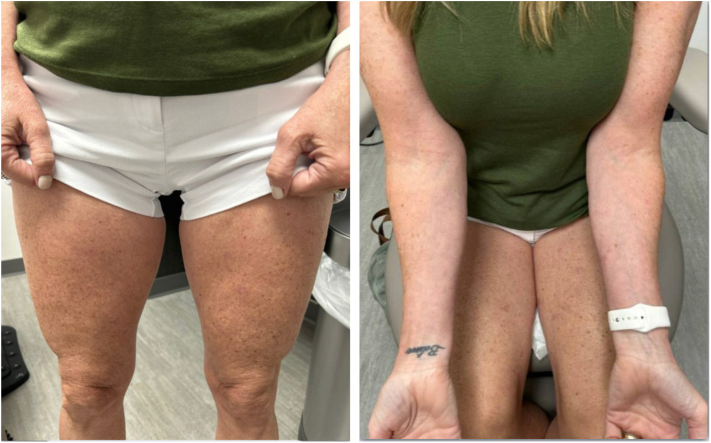
Resolution of TCD with faint hyperpigmented macules and patches after 4 weeks of upadacitinib therapy.

A 54-year-old woman with a history of pityriasis rubra pilaris (PRP) well controlled with guselkumab (IL-23p19 inhibitor) presented with a new 2-week history of a blistering, pruritic rash after clearing trees in the backyard and disposing of them near flora. Physical examination revealed erythematous linear papules, plaques, vesicles, and bullae on the bilateral extremities, with no active lesions of PRP, consistent with a diagnosis of ACD due to poison ivy exposure (TCD) (Fig 1). She had tried OTC calamine lotion and hydroxyzine without sufficient relief. Given the severity of her symptoms and degree of body surface area involvement, the decision was made to forgo topical therapies in favor of systemic therapy. However, given that her PRP initially presented with near-erythroderma and required >2 years of ongoing treatment to clear, there was a strong concern about the potential for rebound flare after a course of prednisone. As a non-steroidal alternative, a tapering course of upadacitinib was prescribed (30 mg for 2 weeks followed by 15 mg for 2 weeks). Although multiple JAK inhibitors were considered, upadacitinb was selected due to availability of the medication. The patient reported that pain and pruritus subsided entirely 24 hours after taking the first dose. At 1-month follow-up, physical exam revealed near-complete resolution of all lesions, with only a few scattered, fading hyperpigmented macules and patches on the bilateral extremities, consistent with resolved ACD/TCD, and no active PRP lesions (Fig 2).

## Discussion

The Janus kinase/signal transducer and activator of transcription (JAK/STAT) pathway is activated when inflammatory cytokines bind their receptors, prompting dimerized receptor-associated JAKs to be autophosphorylated and subsequently to phosphorylate STAT proteins. Activated STAT dimers translocate to the nucleus and regulate transcriptional programs that drive the production of inflammatory cytokines and growth factors.^4^^,^^5^ In dermatology, JAK inhibitors (JAKi) have demonstrated efficacy across several immune-mediated diseases, including alopecia areata, psoriatic arthritis, and AD, by modulating multiple T-cell pathways signaling central to cutaneous inflammation.^4^

ACD, including TCD, is a type IV, T-cell–mediated hypersensitivity in which multiple cytokine axes (eg, IFN-γ/IL-12 for Th1, IL-4/IL-13 for Th2, and IL-17/IL-23 for Th17) contribute to sensitization and elicitation. Given the underlying pathophysiology of ACD reactions, JAK is a mechanistically plausible option for refractory ACD/TCD.^6^ Early clinical experience remains limited but is encouraging. Abrocitinib (JAK1 inhibitor) 100 mg daily achieved complete remission at 8 months in an airborne plant-related ACD case that was unresponsive to dupilumab (an IL-4α receptor antagonist), consistent with prior work demonstrating differential Th polarization with different allergens, however broadly amenable to JAK blockade.^8^ Additionally, upadacitinib at 15 mg daily was successfully used to treat co-existent ACD and psoriasis in 2 patients with allergies to fragrance mix 1 and balsam of Peru.^9^^,^^10^ Especially in severe ACD cases, such as TCD, current first-line systemic therapy is typically oral prednisone for at least 3 weeks. However, in cases where systemic treatment is warranted and prednisone is insufficient or significant concerns about risks are present, short-term use of JAK inhibitors can be an alternative approach with a more favorable safety profile.

Our patient’s rapid and durable improvement in severe poison ivy dermatitis with upadacitinib is consistent with the underlying pathomechanisms of ACD and upadacitinib's mechanism of action. Attenuation of JAK1-dependent signaling likely reduced downstream STAT-mediated inflammation, thereby decreasing T-cell effector responses in TCD. Responses may vary by allergen and by the individual cytokine milieu, but this case adds to emerging evidence that JAK1 inhibition may be effective when conventional measures fail. Although no adverse events were observed in this case, possibly due to the short duration of upadacitinib treatment in combination with long-term guselkumab, potential risks of combining JAK inhibition with other systemic immunomodulatory treatments should considered as there may be overlapping effects in some immune pathways. Prospective studies are needed to define candidate selection, treatment duration, and monitoring parameters. Until such data are available, individualized risk–benefit assessment remains essential, given class-specific safety considerations.

## Conflicts of interest

Dr Ungar is an employee of Mount Sinai and has received research funds (grants paid to the institution) from: Bristol Myers Squibb, Incyte, Rapt Therapeutics, Pfizer, and Sanofi. He is also a consultant for AbbVie, Arcutis Biotherapeutics, Apogee, Therapeutics Bristol Myers Squibb, Botanix Pharmaceuticals, Castle Biosciences, Ebla Holdco, Fresenius Kabi, Galderma, J&J, Leo Pharma, Lilly, Nektar Therapeutics, Pfizer, Primus Pharmaceuticals, Sanofi, Sun Pharma, UCB, Veradermics, VRG Therapeutics. Dr Talia has served as a consultant for Abbvie, Arcutis Biotherapeutics, Bristol-Meyers Squibb, Calliditas Therapeutics, Galderma, Johnson & Johnson, Leo Pharma, Novartis, Navigator Medicines, Primus Pharmaceuticals, Sanofi Genzyme, Stifel Financial, and UCB. He serves or has served as an investigator for Attovia Therapeutics, LEO Pharma, Priovant Therapeutics, and Sanofi. Tai, Tang, and Levine have no conflicts of interest to declare.
